# Chronic Obstructive Pulmonary Disease Subtypes. Transitions over Time

**DOI:** 10.1371/journal.pone.0161710

**Published:** 2016-09-09

**Authors:** Cristóbal Esteban, Inmaculada Arostegui, Myriam Aburto, Javier Moraza, José M. Quintana, Amaia García-Loizaga, Luis V. Basualdo, Amaia Aramburu, Susana Aizpiri, Ane Uranga, Alberto Capelastegui

**Affiliations:** 1 Respiratory Department. Hospital Galdakao-Usansolo, Galdakao, Bizkaia, Spain; 2 Health Services Research on Chronic Patients Network (REDISSEC), Galdakao, Bizkaia, Spain; 3 Department of Applied Mathematics, Statistics and Operative Research, University of the Basque Country (UPV/EHU), Leioa, Bizkaia, Spain; 4 Basque Center for Applied Mathematics (BCAM), Leioa, Bizkaia, Spain; 5 Research Unit, Hospital Galdakao-Usansolo, Galdakao, Bizkaia, Spain; National and Kapodistrian University of Athens, GREECE

## Abstract

**Background:**

Although subtypes of chronic obstructive pulmonary disease are recognized, it is unknown what happens to these subtypes over time. Our objectives were to assess the stability of cluster-based subtypes in patients with stable disease and explore changes in clusters over 1 year.

**Methods:**

Multiple correspondence and cluster analysis were used to evaluate data collected from 543 stable patients included consecutively from 5 respiratory outpatient clinics.

**Results:**

Four subtypes were identified. Three of them, A, B, and C, had marked respiratory profiles with a continuum in severity of several variables, while the fourth, subtype D, had a more systemic profile with intermediate respiratory disease severity. Subtype A was associated with less dyspnea, better health-related quality of life and lower Charlson comorbidity scores, and subtype C with the most severe dyspnea, and poorer pulmonary function and quality of life, while subtype B was between subtypes A and C. Subtype D had higher rates of hospitalization the previous year, and comorbidities. After 1 year, all clusters remained stable. Generally, patients continued in the same subtype but 28% migrated to another cluster. Together with movement across clusters, patients showed changes in certain characteristics (especially exercise capacity, some variables of pulmonary function and physical activity) and changes in outcomes (quality of life, hospitalization and mortality) depending on the new cluster they belonged to.

**Conclusions:**

Chronic obstructive pulmonary disease clusters remained stable over 1 year. Most patients stayed in their initial subtype cluster, but some moved to another subtype and accordingly had different outcomes.

## Introduction

Grouping patients by their characteristics has long been an aspiration in chronic obstructive pulmonary disease (COPD) [1]. In recent years, with stronger evidence of heterogeneity in COPD [2], the level of interest has increased, and this has seen the creation of groups [3], phenotypes [4], and severity scores [5] in an attempt to determine prognosis, improve the process of care and/or move towards precision medicine in the treatment of the disease. One of these approaches has involved identifying characteristics to classify these patients into clusters. The goal of clustering COPD patients into subtypes may be related to prediction of outcomes (prognosis) or to establishing more effective treatment or care strategies. The information available in this area is scarce but important, some studies focusing on stable COPD patients [6–9] and others on exacerbated patients [10,11].

Current information available about COPD phenotypes in stable COPD using clusters has identified several clusters, but there are three main phenotypes that consistently appear in all the different studies. A group with a high level of respiratory symptoms, severe obstruction, lower exercise capacity and lung density, and a low level of severe comorbidities (mainly diabetes and heart disease), this group being labeled the “severe respiratory phenotype” by Garcia-Aymerich et al. [7] The second phenotype, labeled “systemic COPD,” is associated with considerable comorbidities (cardiovascular diseases and diabetes) but moderate-to-severe airway obstruction. The third phenotype had a lower level of dyspnea, a lower rate of comorbidities, and mild-to-moderate airway obstruction. The aforementioned three clusters are in concordance with those described by Burgel et al. [8] and by Rennard et al. [9]. These clusters are related to important outcomes such as hospitalization [7, 9] and mortality [7–9].

To the best of our knowledge, this process has not been taken further, and to do so we need to know the evolution of these COPD clusters over time. Combining multivariate techniques to analyze clinical data obtained in a prospective cohort study could provide validation of the structure of stable COPD phenotypes and transitions between phenotypes over time. The objective of our study was to assess the stability of cluster-based COPD subtypes in stable patients and to identify any changes in the individuals´ subtypes, that is, transitions between COPD clusters, over 1 year of follow-up.

## Methods

### Participants and data collection

We recruited patients being treated for COPD between January 2003 and January 2004 at one of five outpatient respiratory clinics run by the Respiratory Service of Galdakao-Usansolo Hospital. Patients were consecutively included in the study if they had been diagnosed with COPD for at least 6 months and had been stable for 6 weeks before enrollment. Other inclusion criteria were forced expiratory volume in one second (FEV1) <80% of the predicted value and a FEV1/forced vital capacity ratio <70%.

Patients were not eligible for the study if they had been diagnosed with asthma, they had another important respiratory disease or cancer, or they were suffering from psychiatric or neurological problems that might prevent effective collaboration. The protocol was approved by the Ethics and Research Committees of Hospital Galdakao-Usansolo (03/09062005). Each candidate patient was given detailed information about the study and all those included provided written informed consent.

### Study protocol

Sociodemographic variables and smoking habits were recorded. The level of dyspnea was established using modified Medical Research Council scale (mMRC) [12]. Comorbidities were determined by reviewing patients' medical records and summarized in the Charlson comorbidity index [13]. Health-related quality of life (HRQoL) was assessed using the Spanish validated version of the Saint George's Respiratory Questionnaire (SGRQ) [14,15]. The level of physical activity, defined as the time patients spent walking during their leisure time, was classified into four categories: low, moderate, high, and very high corresponding respectively to engaging in light physical activity such as walking for less than 2 hours/week, for 2 to 4 hours/week, more than 4 hours/week and more than 4 hours/week plus playing sports or doing some other type of intense physical activity [16,17]. Complete pulmonary function tests included forced spirometry, bronchodilator testing, and body plethysmography, as well as measurements of carbon monoxide diffusing capacity, and respiratory muscle strength. These tests were performed following the standards of the Spanish Society of Respiratory Medicine and Thoracic Surgery (SEPAR) [18]. For theoretical values, we considered those established by the European Community for Steel and Coal [19].

Two 6-minute walking tests were performed according to American Thoracic Society guidelines [20]. Peripheral muscle strength was evaluated in terms of handgrip strength [21] and extension knee force and shoulder abduction using a hand-held dynamometer [22].

### Follow-up

One year after inclusion in the study, survivors were interviewed and repeated the aforementioned assessments. No interventions were performed related to this study, and the research team did not take part in patients' routine treatment that mainly include long acting bronchodilators and inhaled corticosteroids, or the treatment of any exacerbations.

During this 2-year follow-up period, patient medical records and the hospital database on hospitalizations were reviewed. Vital status was established by reviewing medical records, examining the hospital database and public death registries. Deaths were considered confirmed if the name, sex, and date of birth on the record matched those of the participant.

### Statistical analysis

We combined multiple correspondence analysis (MCA) and cluster analysis to analyze clinical data on all participants included in the study [23,24]. Appendix in the Supporting Information shows a brief description on MCA and cluster analysis, including interpretation of the results (S1 File). MCA was performed twice: first, baseline characteristics were used to define the phenotype structure, and second, data for the same cohort 1 year later were added to the baseline data in order to identify any transitions between phenotypes. Variables included in the MCA, along with the corresponding categories, are listed in the Online Data Supplement, (S1 Table). Cluster analysis was performed using the relative position of the categories given by the MCA factors, the health components initially provided by the MCA being used to identify groups of individuals [25]. The number of groups was selected based on minimum loss of inertia [26]. Participants were classified using the health components at baseline, identifying cluster-based COPD phenotypes. Reclassification after 1 year was performed with both baseline and follow-up health components, showing the transition of participants across the COPD groups during the follow-up period. Phenotype structure stability was assessed by comparing the individual results from the two time points. Phenotypes were validated by assessing their relationships with key outcomes such as mortality, hospitalization, quality of life and COPD multidimensional severity scores. Association of cluster with outcomes was performed by chi-squared test for mortality, hospitalizations and GOLD classification; whereas analysis of variance was performed for HRQoL and severity scores (HADO-AH, BODE and ADO). Kaplan-Meier survival curves stratified by clusters were calculated. All statistical analyses were performed using R v2.13.0 [27].

## Results

The cohort included 543 patients, of whom 96% were male. The cohort had a mean age of 68.3 (SD ± 8.3) years, body mass index of 28.3 (SD ± 4.4) kg/m^2^, mMRC dyspnea score of 2.4 (SD ± 0.9), post-bronchodilator FEV1% of 55% (SD ± 13.3) of the predicted value, and 6-minute walking distance of 408.9 m (SD ± 92.4).

### MCA analysis

**Fig 1:**
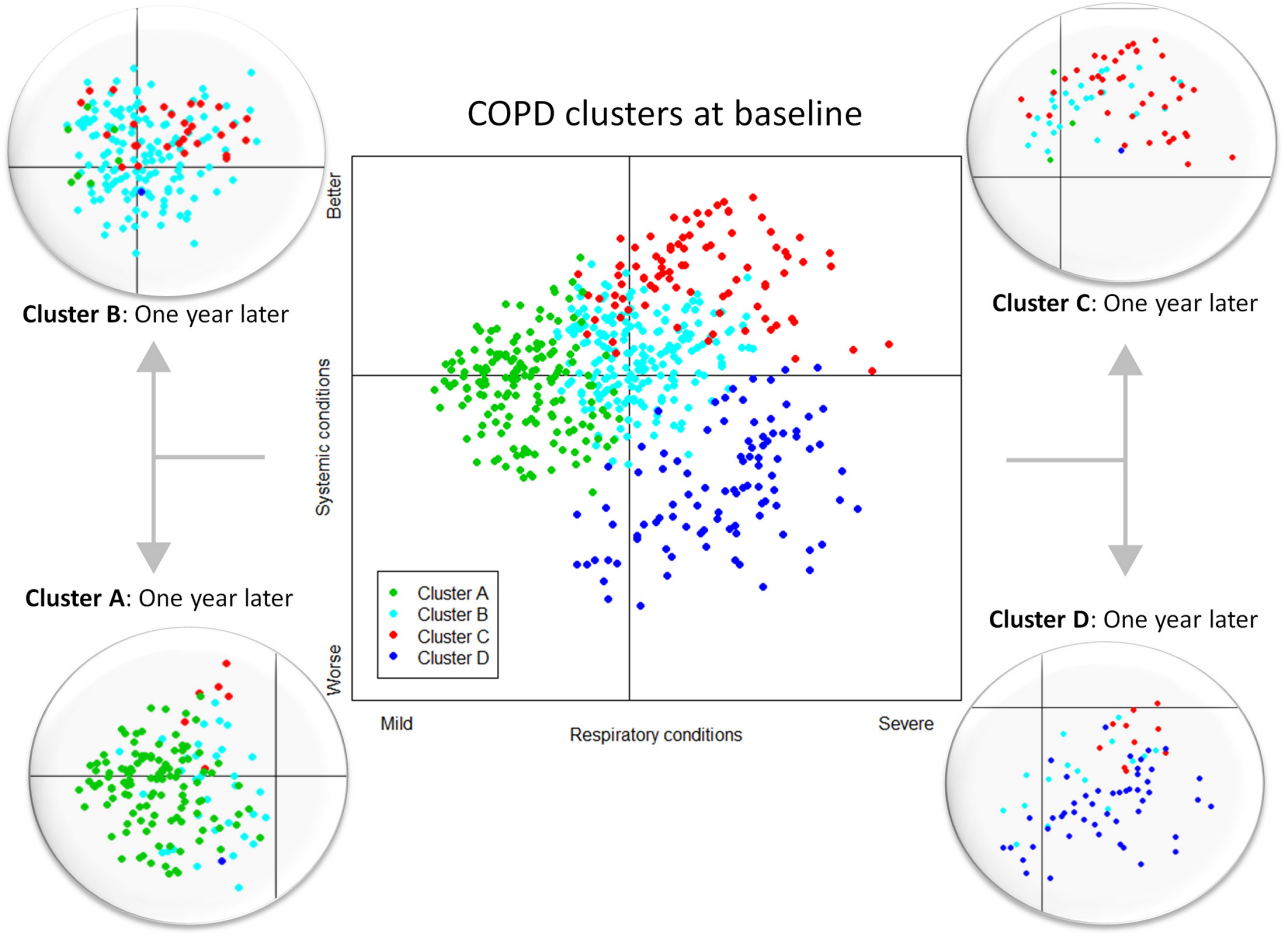
Map of clusters and distribution of patients. Map created by the first and second components derived from the MCA is shown at the center. Four circles at the sides show how patients move between clusters after one year of follow-up. Relative positions of the subjects in the planes are represented by different colors, depending on the subtype provided by the cluster analysis. Definition of the axes is suggested based on information provided in appendix Table A1. The horizontal axis, first component, can be defined as an index of the respiratory conditions of the patient, milder (left side) vs. more severe (right side). The vertical axis, second component, can be defined as an index of the systemic status, worse (bottom) vs. better (top).

Results from the MCA showed that 73.1% of the variance in the original variables was explained by four factors when analysis was performed at baseline and a similar figure of 73.3% was obtained for analysis of the variables at 1 year. Results were almost identical for the two periods of time, showing the high stability of the variables that contribute to the loading factors obtained from the MCA. Patient groupings can be shown graphically considering the two main factors (Fig 1). Categories that significantly contributed to the first factor were age < 65 years, FEV1% ≥ 50%, hand strength ≥ 40 kg, 6-minute walking distance ≥ 425 m, a very high level of physical activity and dyspnea score of 0–1 on the negative side; and FEV1% < 30%, hand strength < 30 kg, 6-minute walking distance < 350 m, a low-to-moderate level of physical activity and dyspnea score ≥ 2 on the positive side. Therefore, we have labeled this first factor “respiratory condition” (graphically represented by the horizontal axis, going from a mild impairment on the negative part of the axis to a severe impairment on the positive part).

The second factor was based mainly on body mass index ≥ 30 kg/m^2^, Charlson comorbidity score > 3 and presence of comorbidities on the negative side and by body mass index < 25 kg/m^2^, Charlson comorbidity score 0–1 and no comorbidities on the positive side. Therefore, we have labeled the second factor “systemic condition”, basically related to number of comorbidities (graphically represented by the vertical axis, with presence of comorbidities on the negative part of the axis and absence of comorbidities on the positive part). Contributions from each variable to the first and second health components is shown in detail in the Supporting Information material (S1 Table). The third and fourth factors were a mixture of many of the original variables, and hence we decided not to give them a specific label.

### Clustering

**Fig 2:**
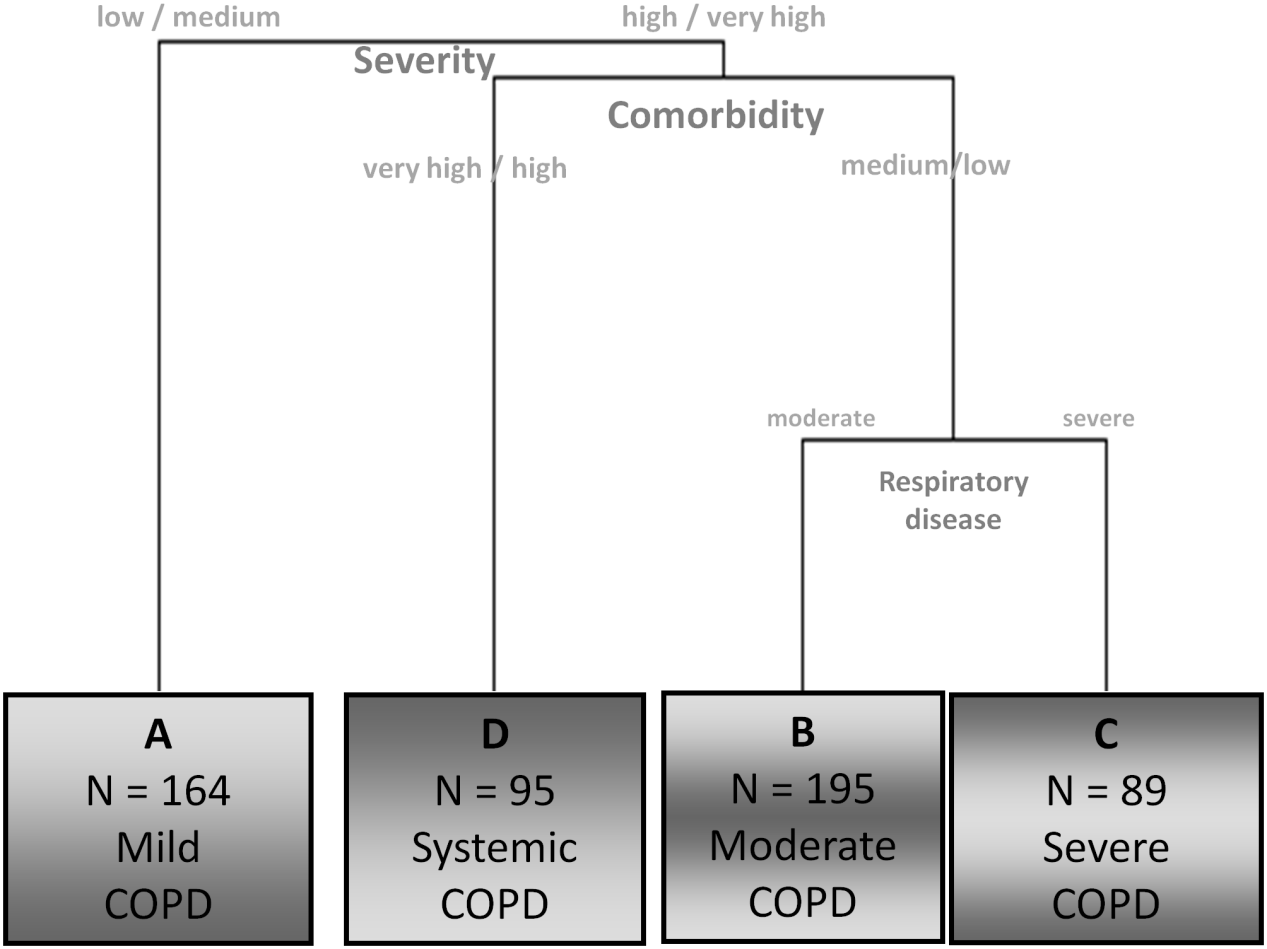
Partial dendrogram obtained from the cluster analysis. The dendogram represents the results from the cluster analysis performed with the four components obtained from the multiple correspondence analysis. The graphical display includes an easy interpretation of the partition and a brief description of the resulting clusters.

In the clustering process (Fig 2), patients were first classified based equally on the respiratory and systemic conditions (first and second factors). At that point, it was the comorbidity status that helped discriminate between two subtypes of patients (second factor). Subsequently, participants with fewer comorbidities were split again into two clusters based on their respiratory impairment (first factor). Third and fourth factors contribute at all three splitting points, although to a lesser extent. The four subtypes (A, B, C, and D) provide a typology of stable COPD patients.

Subtype A included younger participants, with less dyspnea, higher diffusing capacity of the lung for carbon monoxide (DLCO), muscle strength, exercise capacity, and level of physical activity, better HRQoL and lower Charlson comorbidity scores (Tables 1 and 2). Those with subtype C had the heaviest smoking history, highest dyspnea scores, poorest pulmonary function and HRQoL, and lowest muscle strength, exercise capacity, and level of physical activity. However, this subtype was associated with a similar Charlson comorbidity score to subtype A. In relation to the aforementioned variables, patients in subtype B are between subtypes A and C, and had very similar characteristics to subtype D regarding respiratory condition. However, apart from more overall comorbidities, patients in subtype D had a higher rate of hospitalization the year before, and lower exercise capacity and level of physical activity than those in subtype B (p<0.05). As regard as treatment, the only statistically significant difference between clusters was that cluster A shows a lower use of long-acting bronchodilator medication (LABA and/or LAMA) than the rest of the clusters. Our cluster classification matched the Global Strategy for Diagnosis, Management, and Prevention of COPD (GOLD) classification for subtypes A and C. In contrast, participants with subtype D were distributed in similar proportions across every GOLD category, and those with subtype B were also spread across the GOLD categories (though somewhat less evenly). With respect to the relationship of our clusters and multidimensional severity scales, the cluster classification showed a good association with all the multidimensional scales studied. However, the BODE index allowed to establish a different prognosis in every cluster, whereas neither the ADO nor the HADO-AH were able to differentiate between the cluster B and D prognosis (Table 3).

**Table 1:** Distribution of the main clinical and functional variables related to clusters at baseline.

	Cluster A	Cluster B	Cluster C	Cluster D	p
	(n = 164)	(n = 195)	(n = 89)	(n = 95)	
Age	63.4 (62.0–64.8) ^B,C,D^	71.1 ^A^ (70.3–72.0)	69.9 (68.2–71.6) ^A^	69.6 (68.0–71.3) ^A^	<0.001
Body mass index, kg/m^2^	28.3 (27.7–28.9) ^C^	28.4 (27.8–29.0)	26.7 (25.6–27.7) ^A^	29.6 (28.5–30.7)	0.006
Smoking (pack years)	42.3 (38.9–45.7) ^C^	45.7 (41.8–49.6) ^C^	53.9 (47.5–60.3) ^A,B,D^	50.4 (44.3–56.6) ^C^	<0.001
Hospital admissions the previous year:	0.09 (0.04–0.14) ^C,D^	0.27 (0.18–0.35) ^C,D^	1.38 (0.94–1.83) ^A,B^	0.94 (0.58–1.29) ^A,B^	<0.001
• 0	150 (91.5)	158 (81.0)	41 (46.1)	53 (55.8)	
• 1–2	14 (8.5)	34 (17.4)	32 (36.0)	33 (34.7)	
• ≥ 3	0	3 (1.5)	16 (18.0)	9 (9.5)	
FEV1%	59.5 (57.8–61.3) ^C^	56.2 (54.4–58.0) ^C^	43.0 (40.3–45.6) ^A,B,D^	56.0 (53.6–58.4) ^C^	<0.001
RV%	156 (150–161) ^C^	155 (149–161) ^C^	183 (170–197) ^A,B,D^	150 (140–160) ^C^	<0.001
DLCO%	83.4 (80.1–86.6) ^B,C,D^	71.7 (68.7–74.8) ^A,C^	51.0 (46.6–55.4) ^A,B,D^	68.7 (64.6–72.8) ^A,C^	<0.001
DLCO/VA%	105 (100–109) ^B,C,D^	96.0 (92.1–99.9) ^A,C^	76.4 (69.9–82.9) ^A,B,D^	94.1 (88.4–99.8) ^A,C^	<0.001
Hand strength, Kg	39.4 (38.0–40.7) ^B,C,D^	32.5 (31.3–33.7) ^A,C^	29.1 (27.3–33.7) ^A,B,D^	33.0 (31.1–34.8) ^A,C^	<0.001
Quadriceps strength, Kg	33.6 (32.4–34.8^) B,C,D^	27.6 (26.5–28.7^) A,C^	23.0 (21.4–24.6^) A,B^	25.7 (24.1–27.3^) A^	<0.001
Shoulder strength, Kg	19.6 (19.0–20.9) ^B,C,D^	17.0 (16.5–17.6) ^A,C^	14.7 (13.8–17.6) ^A,B,D^	16.6 (15.5–17.6) ^A,C^	<0.001
Physical activity					<0.001
• <2 hours/week	0	5 (2.6)	31 (34.8)	12 (12.6)	
• 2–4 hours/week	11 (6.7)	31 (15.9)	39 (43.8)	29 (30.5)	
• >4 hours/week	22 (13.4)	146 (74.9)	16 (18.0)	35 (36.8)	
• >4 hours/week + intense physical activity	131 (79.9)	13 (6.7)	3 (3.4)	19 (20.0)	
6 min. walking test, m	484 (475–493) ^B,C,D^	403 (394–413) ^A,C,D^	340 (319–361) ^A,B^	356 (339–373) ^A,B^	<0.001
Dyspnea (mMRC)	1.86 (1.76–1.96) ^B,C,D^	2.35 (2.25–2.46) ^A,C^	3.28 (3.10–3.50) ^A,B,D^	2.48 (2.32–2.64) ^A,C^	<0.001

Mean (95% CI) for continuous variables and n (%) for categorical variables.

Statistically significant differences between clusters are indicated with the corresponding superscript.

Dyspnea (mMRC): modified Medical Research Council Dyspnea Scale.

RV: residual volume. DLCO: diffusion lung capacity for carbon monoxide. VA: alveolar volume

**Table 2:** Distribution of the comorbidity variables related to clusters at baseline.

Comorbidity: n(%)	Cluster A	Cluster B	Cluster C	Cluster D	p*
	(*n* = 164)	(*n* = 195)	(*n* = 89)	(*n* = 95)	
Myocardial infarction	2 (1.2)	7 (3.6)	1 (1.1)	22 (23.2)	<0.001
Heart failure	5 (3.1)	11 (5.6)	15 (16.9)	48 (50.5)	<0.001
Angina	4 (2.4)	11 (5.6)	2 (2.3)	23 (24.2)	<0.001
Heart arrhythmia	6 (3.7)	16 (8.2)	6 (6.7)	45 (47.4)	<0.001
Valvular heart disease	2 (1.2)	3 (1.5)	1 (1.1)	11 (11.6)	<0.001*
Hypertension	37 (22.6)	89 (45.6)	17 (19.1)	68 (71.6)	<0.001
Peripheral vascular disease	4 (2.4)	11 (5.6)	2 (2.3)	32 (33.7)	<0.001
Cerebrovascular disease	6 (3.7)	16 (8.2)	2 (2.3)	15 (15.8)	<0.001
Gastric ulcer	11 (6.7)	31 (15.9)	12 (13.5)	21 (22.1)	0.004
Liver disease	6 (3.7)	10 (5.1)	3 (3.4)	17 (17.9)	<0.001
Diabetes	14 (8.5)	26 (13.3)	6 (6.7)	42 (44.2)	<0.001
Renal Insufficiency	1 (0.6)	0	0	7 (7.4)	<0.001*
Joint diseases	62 (37.8)	103 (52.8)	45 (50.6)	46 (48.4)	0.033
Spine injuries and disorders	72 (43.9)	94 (48.2)	47 (52.8)	42 (44.2)	0.523
Psychiatric disorders	16 (9.8)	20 (10.3)	11 (12.4)	19 (20.0)	0.071
Charlson comorbidity index	1.61 (1.49–1.74) ^B,D^	2.22 (2.10–2.33) ^A,C,D^	1.75 (1.56–1.94) ^B,D^	4.78 (4.55–5.00) ^A,B,C^	<0.001
• 0–1	92 (56.1)	36 (18.5)	44 (49.4)	0	
• 2–3	69 (42.1)	153 (78.5)	41 (46.1)	1 (1.1)	
• >3	3 (1.8)	6 (3.1)	4 (4.5)	94 (98.9)	

* P values are from the chi-square test, except for valvular heart disease and renal insufficiency, where Fisher’s exact test was used, and Charlson comorbidity index where ANOVA was used.

Statistically significant differences between clusters are indicated with the corresponding superscript

**Table 3:** Association of cluster-based classification to GOLD classification and COPD severity score (HADO-AH, BODE and ADO).

	Cluster-based classification					
	Cluster A	Cluster B	Cluster C	Cluster D	Total	P^¥^
n (%)	n = 164 (30.2)	n = 195 (35.9)	n = 89 (11.4)	n = 95 (17.5)	n = 543	
GOLD						< 0.001
A	121 (73.8)	72 (36.9)	9 (10.1)	28 (29.5)	230 (42.4)	
B	14 (8.5)	40 (20.5)	7 (7.9)	24 (25.3)	85 (15.7)	
C	26 (15.9)	44 (22.6)	11 (2.4)	22 (23.2)	103 (19.0)	
D	3 (1.8)	39 (20.0)	62 (69.7)	21 (22.1)	125 (23.0)	
p*	<0.001	0.002	<0.001	0.751		
HADO-AH	9.07 ^B,C,D^ (8.44–9.71)	14.82 ^A,C^ (14.30–15.34)	19.47 ^A,B,D^ (18.57–20.37)	15.35 ^A,C^ (14.47–16.23)		<0.001
BODE	1.70 ^B,C,D^ (1.53–1.87)	2.69 ^A,C,D^ (2.50–2.88)	4.93 ^A,B,D^ (4.54–5.33)	3.21 ^A,B,C^ (2.92–3.51)		<0.001
ADO	2.63 ^B,C,D^ (2.48–2.79)	3.92 ^A,C^ (3.78–4.06)	5.04 ^A,B,D^ (4.73–5.36)	3.92 ^A,C^ (3.67–4.17)		<0.001

GOLD: Global Initiative for Chronic Obstructive Lung Disease classification.

HADO-AH: Health, Physical Activity, Dyspnea, Obstruction, Age, and Hospitalization score.

BODE: Body Mass Index, Airflow Obstruction, Dyspnea, and Exercise Capacity index.

ADO: Age, Dyspnea, Obstruction index.

* Column-percentages of individuals are shown. P values from chi-square test for equality of proportions are shown for each column.

^¥^ Chi-square test was used for GOLD groups and ANOVA for the rest. Statistically significant differences between clusters are indicated with the corresponding superscript

### Clusters and patients transition and outcomes

Performing cluster analysis for the two periods 1 year apart showed stability of the initial subtypes. When this analysis was performed first with baseline health components and later with both, baseline and follow-up assessments, we observed that movement of participants from one group to another was associated with changes in variables related to their respiratory condition (pulmonary function, exercise capacity, and physical activity) and also, to a lesser extent, the fact that baseline variables placed them on the border between two subtypes and hence small changes in some of variables analyzed moved them from one cluster to another. Transition of patients between clusters over time is shown graphically in Fig 1 and numerically in Table 4.

**Table 4:** Mortality rate, hospitalizations and HRQoL for patients with COPD exacerbation based on the clusters at baseline (1 year period) and based on the clusters transition for a 1 year period.

Baseline		First year		1 year later		Second year	
Cluster	HRQoL*	Mortality	Hospitalizations	Cluster	HRQoL*	Mortality	Hospitalizations
		n (%)	n (%)			n (%)	n (%)
A (n = 164)	29.2 ^B,C,D^ (26.7–31.8)	2 (1.2)	16 (9.8)	A (n = 116)	28.0 (24.4–31.5)^C^	0 (--)	6 (5.2)
				B (n = 34)	28.9 (23.6–34.3)	0 (--)	3 (8.9)
				C (n = 6)	47.4 (27.6–67.2)^A^	1 (16.7)	1 (16.7)
				D (n = 1)	(--)	(--)	(--)
				P^¥^	0.05^£^	0.045	0.295
B (n = 195)	40.0 ^A,C^ (37.4–42.7)	8 (4.1)	35 (17.9)	A (n = 7)	29.7 (12.4–47.1)^C^	0 (--)	1 (2.4)
				B (n = 140)	36.7 (33.8–39.6)^C^	5 (3.6)	18 (12.9)
				C (n = 29)	48.4 (41.4–55.4)^A,B^	7 (24.1)	11 (37.9)
				D (n = 1)	(--)	(--)	(--)
				P^¥^	0.003^£^	0.003	0.012
C (N = 89)	55.1 ^A,B,D^ (51.2–59.1)	11 (12.4)	33 (37.1)	A (n = 3)	66.9 (22.2–111.6)	0 (--)	0 (--)
				B (n = 26)	50.1 (41.4–58.9)	0 (--)	8 (30.8)
				C (n = 41)	53.0 (46.7–59.3)	7 (17.1)	18 (43.9)
				D (n = 1)	(--)	(--)	(--)
				P^¥^	0.406^£^	0.117	0.356
D (n = 95)	39.6 ^A,C^ (35.7–43.5)	16 (16.8)	35 (36.8)	A (n = 0)	(--)	(--)	(--)
				B (n = 16)	32.6 (23.7–41.5)	0 (--)	4 (25.0)
				C (n = 10)	52.0 (36.2–67.9)	4 (40.0)	6 (60.0)
				D (n = 49)	36.5 (30.7–42.3)	7 (14.3)	10 (20.4)
				P^¥^	0.05	0.021	0.039
P^¥^	<0.001	<0.001	<0.001				

* HRQoL is measured with the total dimension score of the St. George´s Respiratory Questionnaire

^¥^ Chi-square test for mortality and hospitalizations and ANOVA for HRQoL are shown. Statistically significant differences between clusters are indicated with the corresponding superscript

^£^ Cluster D is excluded from the pairwise comparisons.

Results of groups with only one individual were not included.

More complete information about characteristics of patients in the clusters over time is provided in the Supporting Information material (S2–S5 Tables).

**Fig 3:**
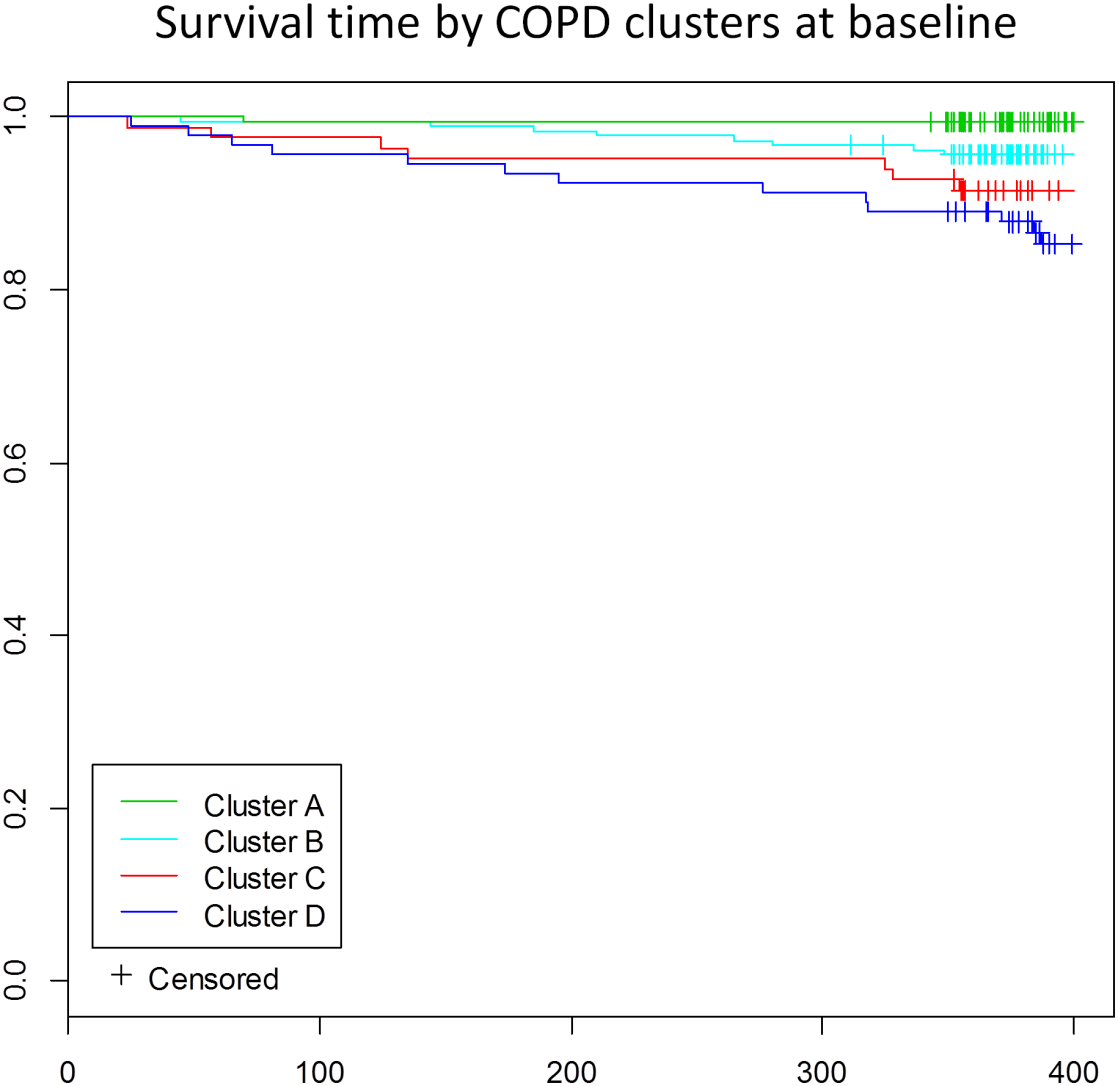
Kaplan-Meier estimate of the survival function during the one year period of follow-up stratified by cluster. Log-rank test for homogeneity (p < 0.001). Significant differences adjusted for multiple comparisons (Bonferroni) were observed between clusters A and C; A and D; and B and D.

Regarding the relation of the four clusters with specific outcomes, significant differences were found for mortality rate, HRQoL scores and hospitalization due to COPD (Table 4). On inclusion in the study, HRQoL was poorest for subtype C, and best for subtype A, with subtypes B and D showing intermediate impairment with similar scores, but significantly different from the two other subtypes. Mortality and hospitalization rates during the first year of follow-up tended to grow from subtype A to subtype D. Regarding as transition of patients between clusters at 1 year, in general those who remained in the same cluster maintained their HRQoL score, whereas those who changed subtype had better or poorer HRQoL depending on their new subtype. Similar trend in results was observed regarding as mortality and hospitalization rates during the next year. The clearest example was patients that moved from subtype D to C with significantly higher mortality and hospitalization rates of 40% and 60% and significantly poorer HRQoL than those remaining at cluster D (Table 4). Moreover, significant differences between survival curves were observed between clusters A and C; A and D; and B and D (Fig 3).

## Discussion

Knowing that in previous important studies [7–9] a longitudinal follow-up was carried out, to our knowledge, this is the first time that temporal evolution of COPD patient clusters has been analyzed, and the following were our main findings. 1) The general characteristics of the clusters remained stable over 1 year. 2) Most patients stayed in their initial cluster, but there was a migration of patients between clusters A, B and C. In general, this migration was towards a more severe cluster, although some patients moved to clusters indicating an improvement in their clinical condition. 3) Our cluster classification is in concordance with previously established COPD clusters. 4) Our clusters showed a clear relationship with different relevant COPD outcomes, and patient migration between clusters was associated with changes in outcomes.

Generally speaking, our four subgroups are based on characteristics that are in concordance with previous cluster analysis studies [7–9]. The identification of clusters with very similar characteristics from different cohorts [7,9] consolidates these subtypes for COPD patients, despite the fact that in one study a considerable number of patients (more than 50%) were not included in any specific cluster [9]. Of the four subtypes we have identified, three (A, B, and C) are suitable for describing a growing severity of the respiratory disease (going from mild to severe) and its expression in other spheres like age, muscle strength, physical activity performed, exercise capacity and HRQoL. Our fourth subtype (D) was very similar to the intermediate subtype of the respiratory group (B) from the point of view of severity of the respiratory disease but with a significantly higher rate of comorbidities. Specifically, the factors that differentiated this “more systemic” subtype from the “intermediate respiratory” subtype are a history of more hospitalizations (i.e., higher rate in the previous year) for COPD exacerbation (higher in the systemic subtype), and the distance covered in the 6-minute walking test (lower in the systemic subtype), as well as, evidently, the rate of comorbidities.

The distribution of our subtypes in relation to the GOLD categories shows that the “mild respiratory” subtype (A) were placed mainly in GOLD group A, and the “severe respiratory” subtype (C) mainly in GOLD group D. On the other hand, the “intermediate respiratory” subtype (B) showed a slight tendency to be placed in GOLD group A, but like our “more systemic” subgroup (D) these patients were distributed fairly evenly across all GOLD groups. This could be interpreted as the GOLD combined assessment not being able to reliably categorize COPD patients with an intermediate level of respiratory disease. Other authors found patients with higher rates of comorbidities to be GOLD groups B or D [28], but this was not the case for our patients. In fact, as mentioned above, patients from subtype D were uniformly distributed across the four GOLD groups. Therefore, it seems that the GOLD categorization does not properly take into account the importance of comorbidities.

We did not find an association between the level of impairment of pulmonary function and the rate of comorbidities, unlike in a previous study [29], patients with the highest level of comorbidities in our cohort being mainly grouped in a cluster with an intermediate level of pulmonary deterioration. When we studied the relationship between our cluster classification and three multidimensional severity scales, we found it to be most strongly correlated with the BODE index, [5], in fact it was the only multidimensional severity scale that was able to capture significant differences between clusters B and D.

There is growing evidence of the importance of comorbidities in COPD and this is an issue that should be considered in the evaluation of patients with this disease. In fact, comorbidity-based clusters have been described [30], but we need to know the impact of these different clusters on outcomes. Moreover, comorbidities have been included in predictive mortality models and scores in COPD [31] and were able to improve the predictive capacity of the BODE index [32].

Our subtype cluster classification is supported by its relationship with powerful outcomes namely mortality and hospitalization for COPD exacerbation. The “more systemic” subtype and the “respiratory severe” subtype showed the highest mortality rates during the 1 year of follow-up after inclusion in the study, of 16.8% and 12.4% respectively.

Reflecting on the previously described patterns, what is particularly interesting in our study is that cluster assignment was not static over the 1-year follow-up. Rather, although most patients stayed in their initial cluster, notable movement was seen across the three respiratory clusters (subtypes A, B, and C). During the 1 year follow-up period, 134 patients (28%) moved to another cluster. Specifically, 81 (17%) migrated to a more severe cluster and 53 (11%) to a less severe cluster, that is, far from negligible movement. Those movements were associated with changes in variables of pulmonary function (FEV1 and diffusion lung capacity for carbon monoxide), exercise capacity, muscle strength and physical activity, and it is plausible that this reflects the concept of disease activity, which would also be changeable over time. Previously, Casanova et al. reported that 27% of patients of a cohort had statistically significant changes in FEV1% and/or BODE index over a year [33], though these changes only reflected deterioration in those variables. In turn, in our study, patients who migrated to other clusters changed their initial prognosis in terms of mortality and hospitalization to those of the cluster to which they had moved.

Twenty-six patients of the “more systemic” cluster (D) migrated to the “intermediate (B) and severe (C) respiratory” clusters maintaining a high level of comorbidity, and this could be interpreted as a weakness of the model. However, those who the model placed in the more “severe respiratory” cluster (C) experienced a very substantial impairment in pulmonary function and exercise capacity over the 1-year follow-up. These changes would explain the change in cluster, as well as their hospitalizations and mortality rates shooting up. In other words those initially in the “more systemic” cluster (D) who move to the “severe respiratory” cluster (C) show a marked worsening in their prognosis. On the other hand, those who migrate from the “more systemic” subtype (D) to the “intermediate respiratory” cluster (B) showed an improvement in exercise capacity and physical activity and, while Charlson comorbidity scores remained steady, the specific comorbidities were very different, those who stayed in the “more systemic” cluster (D) having a higher level of cardio-vascular comorbidity. This pattern could mean that not all comorbidities have the same importance and probably a ranking of impact on prognosis should be established for different comorbidities.

In general terms, some variables repeatedly appeared to be associated with migration between clusters, whatever the direction of the change (better or worse). This could be because changes in 6-minute walking distance over the year were associated with outcomes such as mortality independently of changes in FEV1 [34], while changes in physical activity have been related to hospitalizations and changes in HRQoL [35,36], and muscle strength predicted mortality in a cohort with moderate-to-severe COPD [37]. However, the design of our study does not enable us to establish whether changes in these variables are the cause or the consequence of the migration of patients between clusters.

This study has several limitations. The clinical condition of the three respiratory clusters (A, B, and C) must be understood as a continuum. Although this continuum could be initially attributable to age, in our opinion there are other patients’ conditions that weigh more than age. This assertion is based on the fact that several conditions, not only respiratory, but also muscle strength, physical activity performed or exercise capacity had higher contribution to the first factor than age. Moreover, this continuum means that some patients lie on the border between clusters. Accordingly, small changes in their clinical condition would partially explain changes in classification, although this is not the main reason for the migration between clusters. Further, given the relatively small total number of patients for whom we had complete data for the second clustering process, relatively small numbers of patients migrated to other clusters and hence some results showed interesting tendencies but were not statistically significant. Our cohort had a small proportion of women, because the smoking trend of women in our country, there after the generalizability of the result to other cohort could be controversial. Getting a CT scan of every patient would have enriched our clustering process. In its place we have used the CO transfer as a subrogated marker of emphysema. In fact the CO transfer was one of the variables most frequently involved in the clustering and in the changes of the patient over the follow-up.

On the other hand, the types of analysis performed are a strength of the study. Multivariate techniques combined with cluster analysis have been widely used to differentiate groups of individuals, including for COPD [6–9]. Cluster analysis has previously been used in the literature to classify individuals into groups and also to assess phenotypes in patients with airway diseases [38,39]. Some studies in patients with airway diseases have combined factor and cluster analysis [5]. A strong assumption of factor analysis is that variables included must be continuous and normally distributed. MCA provides an alternative to factor analysis for categorical variables, without any distributional assumptions, and hence was more suitable for our study, that includes patient characteristics that were dichotomous (yes/no) or had three or four discrete possibilities, such as FEV1%, physical activity, and mMRC dyspnea score. MCA has been used previously with similar objectives to ours, including in relation to COPD, when continuous and qualitative explanatory variables were both candidate variables for a model [11]. Thus, we used a methodology previously shown to be useful for the creation of subtypes of individuals and describe their typology for transforming the information contained in the original categorical variables into two factors, which were interpreted as disease components.

In summary, our data allow us to demonstrate the stability of COPD clusters over time and understand the course of COPD patients over a short period of time. During this period, most patients continued to show the same phenotype, but some of them changed their situation, improving or worsening, their condition and therefore their outcomes. These changes should make us consider that we have to improve the characterization of COPD patients and identify these “moving” patients to try to avoid or slow progression of the disease and improve patients’ condition.

## Supporting Information

S1 File Multiple Correspondence Analysis (MCA) and Cluster Analysis (CA). (DOCX)

S1 Table Absolute contribution of each variable by category to the first and second factors of the MCA. ^1^ Indicates the contribution of each category/ level of the variable to that factor or health component, higher value indicates higher contribution. ^2^ Sign indicates the side of the map where each category of each variable is located (referring to Fig 1). The most important contributions along with their signs are highlighted.
(DOCX)

S2 Table Distribution of the main variables related to patient’s COPD at baseline for patients in cluster A (n = 164) and evolution in a one year period including cluster transition. *5 patients (3%) were lost during the follow-up. Confidence intervals for groups of less than 5 individuals were not calculated (showed as --). Mean (95% CI) for continuous variables and n (%) for categorical variables. Dyspnea (mMRC): modified Medical Research Council Dyspnea Scale. RV: residual volume. DLCO: diffusion lung capacity for carbon monoxide. VA: alveolar volume.
(DOCX)

S3 Table Distribution of the main variables related to patient’s COPD at baseline for patients in cluster B (n = 195) and evolution in a one year period including cluster transition. *10 patients (5.1%) were lost during the follow-up. Confidence intervals for groups of less than 5 individuals were not calculated (showed as --). Mean (95% CI) for continuous variables and n (%) for categorical variables. Dyspnea (mMRC): modified Medical Research Council Dyspnea Scale. RV: residual volume. DLCO: diffusion lung capacity for carbon monoxide. VA: alveolar volume.
(DOCX)

S4 Table Distribution of the main variables related to patient’s COPD at baseline for patients in cluster C (n = 89) and evolution in a one year period including cluster transition. *7 patients (7.9%) were lost during the follow-up. Confidence intervals for groups of less than 5 individuals were not calculated (showed as --). Mean (95% CI) for continuous variables and n (%) for categorical variables. Dyspnea (mMRC): modified Medical Research Council Dyspnea Scale. RV: residual volume. DLCO: diffusion lung capacity for carbon monoxide. VA: alveolar volume.
(DOCX)

S5 Table Distribution of the main variables related to patient’s COPD at baseline for patients in cluster D (n = 95) and evolution in a one year period including cluster transition. *4 patients (4.2%) were lost during the follow-up. Confidence intervals for groups of less than 5 individuals were not calculated (showed as --). Mean (95% CI) for continuous variables and n (%) for categorical variables. Dyspnea (mMRC): modified Medical Research Council Dyspnea Scale. RV: residual volume. DLCO: diffusion lung capacity for carbon monoxide. VA: alveolar volume.
(DOCX)
