# Supplementary material for: Chronic Obstructive Pulmonary Disease Subtypes. Transitions over Time
Source: PLoS One. 2016 Sep 9;11(9):e0161710. doi: 10.1371/journal.pone.0161710 (PMC5017635; doi:10.1371/journal.pone.0161710)
Supplement: S1 File — (DOCX) [file pone.0161710.s001.docx]

**Appendix: Multiple Correspondence Analysis (MCA) and Cluster Analysis (CA)**

Multivariate techniques are primarily used in order to synthesize the information contained in a large set of explanatory variables into a few components, often called factors. Such factors retain as much of the information contained in the original set of variables as possible [23]. Some multivariate techniques, such as principal component analysis, are designed exclusively for continuous explanatory variables, whereas others, such as multiple correspondence analysis (MCA), are designed for qualitative explanatory variables [24].

MCA provides descriptive patterns based on categories of the original variables. Each factor provided by this analysis is interpreted and labeled as a component of the health status derived from these original variables. Interpretation of the results is based on graphs or maps of any two factors. The relative position of the category points in the maps indicates the level of similarity or association between the categories: the closer the points, the stronger the relationship between them. A more detailed description of MCA and applications in the health sciences can be consulted elsewhere [24]. Outcome variables that are not specifically included in the model can subsequently be added to the results, by checking their association with the health components identified by the MCA.

Cluster analysis organizes information from apparently heterogeneous individuals into relatively homogeneous groups based on their values in different variables. Moreover, the goal of cluster analysis is to place individuals into groups, such that those in a given group are similar to each other and those in different groups are dissimilar. Cluster analysis is in general designed exclusively for continuous explanatory variables. Groups are not defined a priori, rather they are suggested by the data.

Combining cluster analysis and MCA allows the use of the continuous factors provided by the MCA into the cluster analysis with classification purposes.
