# Supplementary material for: Chronic Obstructive Pulmonary Disease Subtypes. Transitions over Time
Source: PLoS One. 2016 Sep 9;11(9):e0161710. doi: 10.1371/journal.pone.0161710 (PMC5017635; doi:10.1371/journal.pone.0161710)
Supplement: S1 Table — 1 Indicates the contribution of each category/ level of the variable to that factor or health component, higher value indicates higher contribution. 2 Sign indicates the side of the map where each category of each variable is located (referring to Fig 1). The most important contributions along with their signs are highlighted. (DOCX) [file pone.0161710.s002.docx]

S1 Table. Absolute contribution of each variable by category to the first and second

factors of the MCA

|  |  | Contribution^1^ | | | Sign^2^ | |
| --- | --- | --- | --- | --- | --- | --- |
| Variable | Categories | First axes | | Second axes | First axes | Second axes |
| Age | • < 65 | 5 | 0 | | - | - |
|  | • (65, 70) | 0 | 1 | | - | - |
|  | • (70, 75) | 1 | 0 | | + | + |
|  | • ≥ 75 | 2 | 0 | | + | + |
| BMI | • < 25 | 0 | 3 | | - | + |
|  | • (25, 30) | 0 | 0 | | - | + |
|  | • ≥ 30 | 0 | 4 | | + | - |
| Previous  hospitalizations | • 0 | 3 | 0 | | - | - |
|  | • 1 – 2 | 5 | 0 | | + | + |
|  | • ≥ 3 | 5 | 1 | | + | + |
| FEV1% | • < 30 | 3 | 1 | | + | + |
|  | • (30, 50) | 2 | 4 | | + | + |
|  | • ≥ 50 | 2 | 3 | | - | - |
| Hand strength | • < 30 | 3 | 2 | | + | + |
|  | • (30, 35) | 0 | 0 | | + | - |
|  | • (35, 40) | 0 | 1 | | - | - |
|  | • ≥ 40 | 5 | 0 | | - | - |
| Walking test | • < 350 | 13 | 0 | | + | + |
|  | • (350, 425) | 1 | 0 | | + | - |
|  | • (425, 500) | 3 | 0 | | - | - |
|  | • ≥ 500 | 10 | 0 | | - | - |
| Physical  Activity | • Low | 8 | 0 | | + | + |
|  | • Moderate | 3 | 1 | | + | + |
|  | • High | 1 | 1 | | + | - |
|  | • Very high | 15 | 0 | | - | - |
| Dyspnea | • 0 | 4 | 1 | | - | - |
|  | • 1 | 3 | 1 | | - | - |
|  | • 2 | 4 | 0 | | + | + |
|  | • 3 | 3 | 2 | | + | + |
|  | • 4 | 4 | 1 | | + | + |
| Charlson  Comorbidity  Index | • 0 – 1 | 7 | 7 | | - | + |
|  | • 2 – 3 | 0 | 0 | | + | + |
|  | • > 3 | 9 | 17 | | + | - |
| Hypertension | • Yes | 3 | 6 | | + | - |
|  | • No | 2 | 4 | | - | + |
| Diabetes | • Yes | 2 | 7 | | + | - |
|  | • No | 0 | 1 | | - | + |
| Heart attack | • Yes | 2 | 5 | | + | - |
|  | • No | 0 | 0 | | - | + |
| Chronic Heart  Failure | • Yes | 11 | 3 | | + | - |
|  | • No | 2 | 1 | | - | + |
| Angina | • Yes | 2 | 4 | | + | - |
|  | • No | 0 | 0 | | - | + |
| Arrythmia | • Yes | 6 | 7 | | + | - |
|  | • No | 1 | 1 | | - | + |
| Valve disease | • Yes | 1 | 2 | | + | - |
|  | • No | 0 | 0 | | - | + |
| Peripheral  Vascular  Disease | • Yes | 2 | 6 | | + | - |
|  | • No | 0 | 1 | | - | + |
|  |  |  |  | |  |  |

^1^ Indicates the contribution of each category/ level of the variable to that factor or health component, higher value indicates higher contribution. ^2^ Sign indicates the side of the map where each category of each variable is located (referring to Figure 1). The most important contributions along with their signs are highlighted.
