# Supplementary material for: Chronic Obstructive Pulmonary Disease Subtypes. Transitions over Time
Source: PLoS One. 2016 Sep 9;11(9):e0161710. doi: 10.1371/journal.pone.0161710 (PMC5017635; doi:10.1371/journal.pone.0161710)
Supplement: S2 Table — *5 patients (3%) were lost during the follow-up. Confidence intervals for groups of less than 5 individuals were not calculated (showed as --). Mean (95% CI) for continuous variables and n (%) for categorical variables. Dyspnea (mMRC): modified Medical Research Council Dyspnea Scale. RV: residual volume. DLCO: diffusion lung capacity for carbon monoxide. VA: alveolar volume. (DOCX) [file pone.0161710.s003.docx]

S2 Table: Distribution of the main variables related to patient’s COPD at baseline for patients in cluster A (n = 164) and evolution in a one year period including cluster transition.

| n = 164* | Deceased  n = 2 | Cluster A  n = 116 | | Cluster B  n = 34 | | Cluster C  n = 6 | | | Cluster D  n = 1 | |
| --- | --- | --- | --- | --- | --- | --- | --- | --- | --- | --- |
|  |  | Baseline | 1 year | Baseline | 1 year | Baseline | 1 year | | Baseline | 1 year |
| Age | 72 | 62  (60 - 63) | 63  (61 - 65) | 68  (66 – 70) | 69  (67 - 71) | 62  (51 - 73) | 63  (52 - 74) | | 68  -- | 70  -- |
| BMI | 26  -- | 28  (27 - 29) | 28  (28 - 29) | 29  (28 - 30) | 28  (27 - 29) | 26  (20 - 32) | 24  (18 - 30) | | 28  -- | 28  -- |
| Smoking (paq/year) | 26  -- | 41  (37 - 44) | 41  (37 - 44) | 48  (39 - 58) | 49  (40 - 58) | 48  (5 - 91) | 48  (5 - 91) | | 80  -- | 80  -- |
| Previous  Hospitalizations. |  | | | | | | | | | |
| • 0 | 2 (100) | 105 (90 | 106 (91) | 32 (94) | 30 (88) | 5 (83) | | 5 (83) | 1 (100) | 1 (100) |
| • 1-2 | *0* | 11 (10) | 8 (7) | 2 (6) | 3 (8) | 1 (17) | | 1 (17) | *0* | *0* |
| • >=3 | *0* | *0* | 2 (2) | *0* | 1 (3) | *0* | | *0* | *0* | *0* |
| FEV1% | 59  -- | 61  (59 - 63) | 62  (59 - 64) | 56  (52 - 60) | 56  (51 - 61) | 47  (38 - 57) | | 47  (34 - 60) | 44  -- | 69  -- |
| RV% | 166  -- | 156  (149 - 163) | 150  (144 - 157) | 156  (143 - 169) | 159  (140 - 178) | 165  (130 - 201) | | 162  (137 - 187) | 158  -- | 136  -- |
| DLCO% | 56  -- | 86  (53 - 90) | 89  (85 - 93) | 78  (70 - 85) | 80  (71 - 89) | 71  (46 - 97) | | 65  (45 - 86) | 81  -- | 70  -- |
| DLCO/VA | 71  -- | 107  (102 - 112) | 106  (102 - 111) | 102  (92 - 111) | 102  (92 - 112) | 95  (56 - 133) | | 89  (52 - 126) | 88  -- | 80  -- |
| Hand strength | 33  -- | 40  (38 - 42) | 39  (38 - 41) | 39  (37 - 41) | 39  (37 - 40) | 33  (26 - 40) | | 31  (24 - 38) | 38  -- | 38  -- |
| Quadriceps strength | 28  -- | 37  (36 - 38) | 36  (34 - 38) | 34  (31 - 36) | 32  (29 - 34) | 24  (16 - 33) | | 20  (14 - 27) | 45  -- | 45  -- |
| Shoulder strength | 16  -- | 20  (19 - 21) | 19  (18 - 20) | 20  (19 - 21) | 17  (15 - 19) | 16  (12 - 19) | | 13  (10 - 16) | 26  -- | 25  -- |
| Physical activity |  | | | | | | | | | |
| • < 2 hours/week | *0 --* | *0 --* | *0 --* | *0 --* | *0 --* | *0 --* | | 1 (17) | *0 --* | *0 --* |
| • 2-4 hours/week | 0 -- | 3 (3) | 6 (5) | 5 (15) | 5 (15) | 3 (50) | | 1 (17) | 0 -- | 0 -- |
| • >4 hours/week | 0 -- | 12 (10) | 21 (18) | 10 (29) | 21 (62) | 0 -- | | 3 (50) | 0 -- | 0 -- |
| • >4 hours/week +Intense physical activity | 2 (100) | 101 (87) | 89 (77) | 19 (56) | 8 (26) | 3 (50) | | 1 (17) | 1 (100) | 1 (100) |
| 6 min.walking test | 508  -- | 498  (488 - 508) | 516  (502 - 530) | 442  (424 - 460) | 458  (442 - 475) | 465.5  (401 - 530) | | 449  (375 - 523) | 437  -- | 460  -- |
| Dyspnea | 2.5  -- | 1.8  (1.7 - 1.9) | 1.7  (1.6 - 1.8) | 1.9  (1.8 - 2.1) | 1.9  (1.8 - 2.1) | 2.3  (1.5 - 3.2) | | 2.7  (2.1 - 3.2) | 2.0  -- | 1.0  -- |
| Charlson-index | 2.0  -- | 1.5  (1.4 - 1.7) | 1.6  (1.5 - 1.8) | 1.9  (1.6 - 2.1) | 1.9  (1.6 - 2.2) | 1.0  -- | | 1.0  -- | 4.0  -- | 4.0  -- |
| • 0-1 | 1 (50) | 70 (60) | 68 (59) | 13 (38) | 12 (35) | 6 (100) | | 6 (100) | 0 -- | 0 -- |
| • 2-3 | 1 (50) | 45 (39 | 29 (25) | 20 (59) | 15 (44) | 0 -- | | 0 -- | 0 -- | 0 -- |
| • >3 | 0 -- | 1 (1) | 19 (16) | 1 (3) | 7 (21) | 0 -- | | 0 -- | 1 (100) | 1 (100) |
| Peripheral vascular disease | 0 -- | 2 (2) | 3 (3) | 2 (6) | 2 (6) | 0 -- | | 0 -- | 0 -- | 0 -- |
| Diabetes | 0 -- | 9 (8) | 10 (9) | 4 (12) | 4 (12) | 0 -- | | 0 -- | 0 -- | 0 -- |
| Heart disease | 1 (50) | 10 (9) | 10 (9) | 6 (18) | 6 (18) | 0 -- | | 0 -- | 1 (100) | 1 (100) |
|  |  |  |  |  |  |  | |  |  |  |

*5 patients (3%) were lost during the follow-up. Confidence intervals for groups of less than 5 individuals were not calculated (showed as --)

Mean (95% CI) for continuous variables and n (%) for categorical variables.

Dyspnea (mMRC): modified Medical Research Council Dyspnea Scale.

RV: residual volume. DLCO: diffusion lung capacity for carbon monoxide. VA: alveolar volume.
