# Supplementary material for: Chronic Obstructive Pulmonary Disease Subtypes. Transitions over Time
Source: PLoS One. 2016 Sep 9;11(9):e0161710. doi: 10.1371/journal.pone.0161710 (PMC5017635; doi:10.1371/journal.pone.0161710)
Supplement: S3 Table — *10 patients (5.1%) were lost during the follow-up. Confidence intervals for groups of less than 5 individuals were not calculated (showed as --). Mean (95% CI) for continuous variables and n (%) for categorical variables. Dyspnea (mMRC): modified Medical Research Council Dyspnea Scale. RV: residual volume. DLCO: diffusion lung capacity for carbon monoxide. VA: alveolar volume. (DOCX) [file pone.0161710.s004.docx]

S3 Table: Distribution of the main variables related to patient’s COPD at baseline for patients in cluster B (n = 195) and evolution in a one year period including cluster transition.

| n = 195* | Deceased  n = 8 | Cluster A  n = 7 | | Cluster B  n = 140 | | Cluster C  n = 29 | | | Cluster D  n = 1 | |
| --- | --- | --- | --- | --- | --- | --- | --- | --- | --- | --- |
|  |  | Baseline | 1 year | Baseline | 1 year | Baseline | 1 year | | Baseline | 1 year |
| Age | 74  (972 - 76) | 64  (57 - 71) | 65  (58 - 72) | 71  (70 - 72) | 72  (71 - 73) | 71  (68 - 73) | 72  (69 - 74) | | 65  -- | 66  -- |
| BMI | 27  (23 - 30) | 32  (25 - 38) | 31  (25 - 38) | 29  (28 - 29) | 29  (28 - 29) | 28  (26 - 29) | 27  (25 - 28) | | 34  -- | 36  -- |
| Smoking (pack/year) | 45  (21 - 68) | 49  (35 - 64) | 50  (35 - 64) | 46  (41 - 50) | 46  (41 - 50) | 43  (31 - 55) | 43  (32 - 55) | | 75  -- | 75  -- |
| Previous  Hospitalizations. |  | | | | | | | | | |
| • 0 | 7 (87) | 7 (100) | 5 (71) | 113 (81) | 122 (87) | 21 (72) | | 17 (59) | 0 -- | 0 -- |
| • 1-2 | 1 (13) | 0 -- | 2 (29) | 25 (18) | 17 (12) | 7 (24) | | 10 (34) | 1 (100) | 1 (100) |
| • >=3 | 0 -- | 0 -- | 0 -- | 2 (1) | 1 (1) | 1 (4) | | 2 (7) | 0 -- | 0 -- |
| FEV1% | 47  (32 - 62) | 57  (44 - 69) | 63  (54 - 72) | 57  (55 - 59) | 58  (56 - 60) | 54  (49 - 59) | | 46  (37 - 54) | 67 | 67 |
| RV% | 155  (117 - 193) | 162  (125 - 200) | 138  (88 - 188) | 154  (147 - 161) | 156  (149 - 164) | 158  (143 - 174) | | 139  (113 - 166) | 149  -- | 120  -- |
| DLCO% | 60  (47 - 72) | 85  (64 - 105) | 81  (55 - 107) | 75  (71 - 78) | 78  (73 - 82) | 58  (52 - 65) | | 49  (39 - 60) | 97  -- | 81  -- |
| DLCO/VA | 84  (65 - 102) | 103  (76 - 130) | 99  (70 - 128) | 99  (94 - 103) | 96  (92 - 101) | 85  (74 - 95) | | 73  (58 - 87) | 128  -- | 112  -- |
| Hand strength | 29  (25 - 33) | 44  (39 - 49) | 41  (32 - 50) | 33  (31 - 34) | 32  (31 - 33) | 31  (27 - 34) | | 25  (20 - 29) | 58  -- | 53  -- |
| Quadriceps strength | 27  (23 - 31) | 40  (36 - 44) | 35  (16 - 53) | 29  (27 - 30) | 28  (26 - 29) | 24  (21 - 27) | | 18  (14 - 21) | 45  -- | 39  -- |
| Shoulder strength | 15  (12 - 18) | 22  (18 - 26) | 20  (8 - 32) | 17  (16 - 18) | 16  (15 - 17) | 16  (14 - 17) | | 11  (8 - 14) | 36  -- | 28  -- |
| Physical activity |  | | | | | | | | | |
| • < 2 hours/week | 0 -- | 0 -- | 0 -- | 3 (2) | 5 (4) | 2 (7) | | 6 (21) | 0 -- | 0 -- |
| • 2-4 hours/week | 1 (13) | 0 -- | 0 -- | 15 (11) | 29 (21) | 12 (41) | | 11 (38) | 1 (100) | 1 (100) |
| • >4 hours/week | 5 (62) | 6 (86) | 4 (57) | 117 (82) | 94 (67) | 13 (45) | | 11 (38) | 0 -- | 0 -- |
| • >4 hours/week +intense physical activity | 2 (25) | 1 (14) | 3 (43) | 7 (5) | 12 (9) | 2 (7) | | 1 (3) | 0 -- | 0 -- |
| 6 minutes walking test | 379  (326 - 432) | 437  (357 - 517) | 462  (358 - 566) | 409  (398 - 421) | 419  (404 - 433) | 380  (356 - 404) | | 310  (262 - 359) | 276  -- | 478  -- |
| Dyspnea | 2.5  (1.4 - 3.6) | 2.0  (1.2 - 2.8) | 2.0  (1.2 - 2.8) | 2.3  (2.1 - 2.4) | 2.1  (2.0 - 2.2) | 2.9  (2.6 - 3.2) | | 2.7  (2.4 - 3.0) | 1.0  -- | 1.0  -- |
| Charlson-index | 2.8  (2.2 - 3.3) | 1.7  (1.3 - 2.2) | 1.9  (1.2 - 2.5) | 2.2  (2.1 - 2.3) | 2.4  (2.2 - 2.5) | 2.3  (2.0 - 2.5) | | 2.8  (2.4 - 3.2) | 2.0  -- | 2.0  -- |
| • 0-1 | 0 -- | 2 (28.6) | 2 (28.6) | 27 (19.3) | 22 (15.7) | 4 (13.8) | | 2 (6.9) | 0 -- | 0 -- |
| • 2-3 | 7 (87.5) | 5 (71.4) | 4 (57.1) | 108 (77.1) | 58 (41.4) | 25 (86.2) | | 10 (34.5) | 1 (100) | 1 (100) |
| • >3 | 1 (12.5) | 0 -- | 1 (14.3) | 5 (3.6) | 60 (42.9) | 0 -- | | 17 (58.6) | 0 -- | 0 -- |
| Peripheral vascular disease | 1 (13) | 0 -- | 0 -- | 9 (6) | 10 (7) | 1 (4) | | 1 (4) | 0 -- | 0 -- |
| Diabetes | 2 (25) | 0 -- | 0 -- | 22 (16) | 25 (18) | 4 (7) | | 4 (14) | 0 -- | 0 -- |
| Heart disease | 1 (13) | 0 -- | 1 (14) | 28 (20) | 32 (23) | 8 (28) | | 12 (41) | 0 -- | 0 -- |
|  |  |  |  |  |  |  | |  |  |  |

*10 patients (5.1%) were lost during the follow-up. Confidence intervals for groups of less than 5 individuals were not calculated (showed as --).

Mean (95% CI) for continuous variables and n (%) for categorical variables.

Dyspnea (mMRC): modified Medical Research Council Dyspnea Scale.

RV: residual volume. DLCO: diffusion lung capacity for carbon monoxide. VA: alveolar volume.
