# Supplementary material for: Chronic Obstructive Pulmonary Disease Subtypes. Transitions over Time
Source: PLoS One. 2016 Sep 9;11(9):e0161710. doi: 10.1371/journal.pone.0161710 (PMC5017635; doi:10.1371/journal.pone.0161710)
Supplement: S4 Table — *7 patients (7.9%) were lost during the follow-up. Confidence intervals for groups of less than 5 individuals were not calculated (showed as --). Mean (95% CI) for continuous variables and n (%) for categorical variables. Dyspnea (mMRC): modified Medical Research Council Dyspnea Scale. RV: residual volume. DLCO: diffusion lung capacity for carbon monoxide. VA: alveolar volume. (DOCX) [file pone.0161710.s005.docx]

S4 Table: Distribution of the main variables related to patient’s COPD at baseline for patients in cluster C (n = 89) and evolution in a one year period including cluster transition.

| n = 89* | Deceased  n = 11 | Cluster A  n = 3 | | Cluster B  n = 26 | | Cluster C  n = 41 | | | Cluster D  n = 1 | |
| --- | --- | --- | --- | --- | --- | --- | --- | --- | --- | --- |
|  |  | Baseline | 1 year | Baseline | 1 year | Baseline | 1 year | | Baseline | 1 year |
| Age | 73  (68 - 77) | 52  -- | 53  -- | 69  (66 - 72) | 70  (67 - 74) | 70  (68 - 72) | 72  (69 - 74) | | 67  -- | 68  -- |
| BMI | 23  (21 - 26) | 34  -- | 33  -- | 27  (26 - 29) | 27  (25 - 29) | 27  (25 - 28) | 26  (24 - 28) | | 34  -- | 37  -- |
| Smoking (pack/year) | 68  (46 - 90) | 35  -- | 35  -- | 55  (41 - 68) | 55  (41 - 69) | 46  (39 - 54) | 46  (39 - 54) | | 50  -- | 50  -- |
| Previous  Hospitalizations. |  | | | | | | | | | |
| • 0 | 4 (36) | 1 (33) | 3 (100) | 14 (54) | 16 (61) | 17 (41) | | 29 (71) | 1 (100) | 1 (100) |
| • 1-2 | 5 (46) | 2 (67) | 0 -- | 9 (35) | 9 (35) | 15 (37) | | 5 (12) | 0 -- | 0 -- |
| • >=3 | 2 (18) | 0 -- | 0 -- | 3 (11) | 1 (4) | 9 (22) | | 7 (1) | 0 -- | 0 -- |
| FEV1% | 42  (32 - 52) | 51  -- | 68  -- | 45  (40 - 50) | 46  (41 - 51) | 40  (37 - 44) | | 37  (33 - 41) | 45  -- | 52  -- |
| RV% | 186  (141 - 230) | 139  -- | 135  -- | 172  (149 - 195) | 165  (142 - 189) | 201  (178 - 221) | | 187  (161 - 213) | 194  -- | 165  -- |
| DLCO% | 35  (26 - 43) | 79  -- | 86  -- | 63  (53 - 72) | 69  (53 - 85) | 47  (43 - 52) | | 38  (30 - 46) | 64  -- | 76  -- |
| DLCO/VA | 56  (37 - 75) | 103  -- | 101  -- | 91  (75 - 103) | 87  (77 - 98) | 73  (64 - 81) | | 57  (445 - 69) | 91  -- | 96  -- |
| Hand strength | 23  (19 - 26) | 42  -- | 45  -- | 33  (30 - 36)) | 33  (28 - 37) | 28  (25 - 31) | | 26  (23 - 28) | 45  -- | 43  -- |
| Quadriceps strength | 17  (13 - 21) | 40  -- | 43  -- | 28  (25 - 30) | 28  (25 - 30) | 23  (20 - 26) | | 18  (14 - 22) | 34  -- | 43  -- |
| Shoulder strength | 11  (9 - 13) | 22  -- | 26  -- | 16  (15 - 18) | 15  (14 - 16) | 14  (13 - 16) | | 12  (10 - 14) | 20  -- | 24  -- |
| Physical activity |  | | | | | | | | | |
| • < 2 hours/week | 5 (46) | 0 -- | 1 (33) | 4 (15) | 2 (8) | 19 (46) | | 24 (59) | 0 -- | 0 -- |
| • 2-4 hours/week | 3 (27) | 1 (33) | 0 -- | 11 (42) | 10 (39) | 19 (46) | | 15 (37) | 1 (100) | 0 -- |
| • >4 hours/week | 3 (27) | 1 (33) | 1 (33) | 9 (35) | 14 (54) | 3 (7) | | 2 (5) | 0 -- | 0 -- |
| • >4 hours/week +intense physical activity | 0 -- | 1 (33) | 1 (33) | 2 (8) | 0 -- | 0 -- | | 0 -- | 0 -- | 1 (100) |
| 6 minutes walking test | 265  (220 - 309) | 446  -- | 509  -- | 400  (369 - 431) | 416  (381 - 451) | 327  (297 - 357) | | 292  (250 - 333) | 345  -- | 417  -- |
| Dyspnea | 3.5  (2.8 - 4.2) | 3.7  -- | 2.7  -- | 3.1  (2.7 - 3.5) | 2.7  (2.3 - 3.0) | 3.4  (3.1 - 3.7) | | 3.3  (2.9 - 3.6) | 2.0  -- | 2.0  -- |
| Charlson-index | 1.9  (1.4 - 2.5) | 1.3  -- | 1.3  -- | 1.5  (1.2 - 1.8) | 1.7  (1.3 - 2.0) | 1.8  (1.5 - 2.1) | | 2.0  (1.7 - 2.3) | 2.0  -- | 3.0  -- |
| • 0-1 | 4 (36) | 2 (67) | 2 (67) | 15 (58) | 13 (50) | 21 (51) | | 18 (44) | 0 -- | 0 -- |
| • 2-3 | 7 (64) | 1 (33) | 1 (33) | 11 (42) | 10 (38) | 18 (44) | | 9 (22) | 1 (100) | 1 (100) |
| • >3 | 0 -- | 0 -- | 0 -- | 0 -- | 3 (12) | 2 (5) | | 14 (34) | 0 -- | 0 -- |
| Peripheral vascular disease | 0 -- | 0 -- | 0 -- | 1 (4) | 1 (4) | 1 (2) | | 1 (2) | 0 -- | 0 -- |
| Diabetes | 1 (9) | 0 -- | 0 -- | 1 (4) | 1 (4) | 3 (7) | | 4 (10) | 0 -- | 1 (100) |
| Heart disease | 3 (27) | 1 (33) | 1 (33) | 2 (8) | 4 (15) | 7 (17) | | 7 (17) | 0 -- | 0 -- |
|  |  |  |  |  |  |  | |  |  |  |

*7 patients (7.9%) were lost during the follow-up. Confidence intervals for groups of less than 5 individuals were not calculated (showed as --)

Mean (95% CI) for continuous variables and n (%) for categorical variables.

Dyspnea (mMRC): modified Medical Research Council Dyspnea Scale.

RV: residual volume. DLCO: diffusion lung capacity for carbon monoxide. VA: alveolar volume
