# Supplementary material for: Chronic Obstructive Pulmonary Disease Subtypes. Transitions over Time
Source: PLoS One. 2016 Sep 9;11(9):e0161710. doi: 10.1371/journal.pone.0161710 (PMC5017635; doi:10.1371/journal.pone.0161710)
Supplement: S5 Table — *4 patients (4.2%) were lost during the follow-up. Confidence intervals for groups of less than 5 individuals were not calculated (showed as --). Mean (95% CI) for continuous variables and n (%) for categorical variables. Dyspnea (mMRC): modified Medical Research Council Dyspnea Scale. RV: residual volume. DLCO: diffusion lung capacity for carbon monoxide. VA: alveolar volume. (DOCX) [file pone.0161710.s006.docx]

S5 Table: Distribution of the main variables related to patient’s COPD at baseline for patients in cluster D (n = 95) and evolution in a one year period including cluster transition.

| n = 95* | Deceased  n = 17 | Cluster A  n = 0 | Cluster B  n = 16 | | Cluster C  n = 10 | | | Cluster D  n = 49 | |
| --- | --- | --- | --- | --- | --- | --- | --- | --- | --- |
|  |  | Baseline | Baseline | 1 year | Baseline | 1 year | | Baseline | 1 year |
| Age | 74  (71 - 77) |  | 73  (70 - 76) | 74  (71 - 77) | 65  (58 - 72) | 66  (59 - 73) | | 68  (66 - 70) | 69  (67 - 71) |
| BMI | 28  (26 - 31) |  | 28  (27 - 30) | 28  (27 - 30) | 26  (21 - 31) | 26  (21 - 31) | | 31  (30 - 32) | 32  (29 - 34) |
| Smoking (pack/year) | 45  (34 - 57) |  | 53  (29 - 77) | 53  (29 - 77) | 50  (35 - 65) | 51  (36 - 66) | | 51  (44 - 59) | 52  (44 - 59) |
| Previous  Hospitalizations. |  |  |  |  |  |  |  |  |  |
| • 0 | 6 (37) |  | 13 (81) | 10 (62) | 4 (40) | 6 (60) | 28 (57) | | 36 (73) |
| • 1-2 | 8 (50) |  | 3 (19) | 6 (38) | 3 (30) | 3 (30) | 17 (35) | | 13 (27) |
| • >=3 | 2 (13) |  | 0 -- | 0 -- | 3 (30) | 1 (10) | 4 (8) | | 0 -- |
| FEV1% | 55  (48 - 62) |  | 61  (55 - 68) | 63  (57 - 69) | 45  (35 - 55) | 36  (19 - 54) | 56  (54 - 59) | | 58  (54 - 61) |
| RV% | 149  (112 - 185) |  | 158  (137 - 180) | 156  (137 - 175) | 175  (132 - 218) | 169  (112 - 226) | 144  (132 - 155) | | 135  (120 - 150) |
| DLCO% | 62  (52 - 73) |  | 77  (65 - 89) | 74  (66 - 83) | 50  (37 - 62) | 37  (18 - 57) | 70  (65 - 76) | | 68  (62 - 74) |
| DLCO/VA | 88  (70 - 105) |  | 97  (85 - 109) | 96  (83 - 108) | 70  (52 - 90) | 52  (25 - 78) | 97  (90 - 105) | | 93  (84 - 101) |
| Hand strength | 31  (27 - 35) |  | 34  (30 - 37) | 33  (28 - 37) | 26  (18 - 34) | 22  (13 - 31) | 34  (32 - 37) | | 35  (33 - 38) |
| Quadriceps strength | 23  (18 - 27) |  | 28  (24 - 31) | 27  (24 - 31) | 21  (13 - 28) | 16  (7 - 25) | 30  (27 - 32) | | 27  (24 - 31) |
| Shoulder strength | 15  (12 - 17) |  | 16  (14 - 19) | 15  (14 - 17) | 14  (9 - 18) | 10  (5 - 15) | 18  (16 - 19) | | 16  (15 - 18) |
| Physical activity |  |  |  |  |  |  |  |  |  |
| • < 2 hours/week | 4 (25) |  | 0 -- | 0 -- | 3 (30) | 2 (20) | 5 (10) | | 3 (6) |
| • 2-4 hours/week | 7 (44) |  | 2 (13) | 2 (13) | 5 (50) | 5 (50) | 15 (31) | | 18 (37) |
| • >4 hours/week | 5 (31) |  | 10 (62) | 12 (75) | 1 (10) | 2 (20) | 17 (35) | | 18 (37) |
| • >4 hours/week +intense physical activity | 0 -- |  | 4 (25) | 2 (13) | 1 (10) | 1 (10) | 12 (24) | | 10 (20) |
| 6 minutes walking test. | 311  (274 - 349) |  | 371  (333 - 408) | 406  (369 - 443) | 332  (267 - 397) | 285  (190 - 380) | 371  (347 - 395) | | 365  (332 - 398) |
| Dyspnea | 2.8  (2.3 - 3.2) |  | 2.2  (1.9 - 2.5) | 2.1  (1.8 - 2.4) | 2.9  (2.5 - 3.3) | 3.0  (2.7 - 3.3) | 2.4  (2.2 - 2,7) | | 2.2  (2.0 - 2.4) |
| Charlson-index | 5.2  (4.5 - 5.9) |  | 4.2  (4.0 - 4.4) | 4.2  (4.0 - 4.4) | 4.4  (3.9 - 4.9) | 4.5  (4.0 - 5.0) | 4.9  (4.6 - 5.3) | | 5.1  (4.8 - 5.5) |
| • 0-1 | 0 -- |  | 0 -- | 0 -- | 0 -- | 0 -- | 0 -- | | 0 -- |
| • 2-3 | 0 -- |  | 0 -- | 0 -- | 0 -- | 0 -- |  | | 0 -- |
| • >3 | 16 (100) |  | 16 (100) | 16 (100) | 10 (100) | 10 (100) | 48 (98) | | 49 (100) |
| Peripheral vascular disease | 3 (19) |  | 6 (38) | 6 (38) | 2 (20) | 2 (20) | 19 (39) | | 19 (39) |
| Diabetes | 7 (44) |  | 7 (44) | 7 (44) | 4 (40) | 4 (40) | 23 (47) | | 25 (51) |
| Heart disease | 15 (94) |  | 6 (38) | 6 (38) | 7 (70) | 7 (70) | 45 (92) | | 45 (92) |
|  |  |  |  |  |  |  |  | |  |

*4 patients (4.2%) were lost during the follow-up. Confidence intervals for groups of less than 5 individuals were not calculated (showed as --).

Mean (95% CI) for continuous variables and n (%) for categorical variables.

Dyspnea (mMRC): modified Medical Research Council Dyspnea Scale.

RV: residual volume. DLCO: diffusion lung capacity for carbon monoxide. VA: alveolar volume.
